# Magnetic protein aggregates generated by supramolecular assembly of ferritin cages - a modular strategy for the immobilization of enzymes

**DOI:** 10.3389/fbioe.2024.1478198

**Published:** 2024-10-23

**Authors:** Gizem Ölçücü, Bastian Wollenhaupt, Dietrich Kohlheyer, Karl-Erich Jaeger, Ulrich Krauss

**Affiliations:** ^1^ Institute of Bio- and Geosciences IBG-1: Biotechnology, Forschungszentrum Jülich GmbH, Jülich, Germany; ^2^ Institute of Molecular Enzyme Technology, Heinrich Heine University Düsseldorf, Forschungszentrum Jülich GmbH, Jülich, Germany; ^3^ Department of Biochemistry, University of Bayreuth, Bayreuth, Germany

**Keywords:** enzyme immobilization, protein-protein interactions, protein aggregates, biocatalysis, magnetic protein aggregates, MPAs, CatMPAs

## Abstract

**Introduction:**

Efficient and cost-effective immobilization methods are crucial for advancing the utilization of enzymes in industrial biocatalysis. To this end, *in vivo* immobilization methods relying on the completely biological production of immobilizates represent an interesting alternative to conventional carrier-based immobilization methods. This study aimed to introduce a novel immobilization strategy using *in vivo*-produced magnetic protein aggregates (MPAs).

**Methods:**

MPA production was achieved by expressing gene fusions of the yellow fluorescent protein variant citrine and ferritin variants, including a magnetically enhanced *Escherichia coli* ferritin mutant. Cellular production of the gene fusions allows supramolecular assembly of the fusion proteins *in vivo*, driven by citrine-dependent dimerization of ferritin cages. Magnetic properties were confirmed using neodymium magnets. A bait/prey strategy was used to attach alcohol dehydrogenase (ADH) to the MPAs, creating catalytically active MPAs (CatMPAs). These CatMPAs were purified via magnetic columns or centrifugation.

**Results:**

The fusion of the mutant *E. coli* ferritin to citrine yielded fluorescent, insoluble protein aggregates, which are released upon cell lysis and coalesce into MPAs. MPAs display magnetic properties, as verified by their attraction to neodymium magnets. We further show that these fully *in vivo*-produced protein aggregates can be magnetically purified without *ex vivo* iron loading. Using a bait/prey strategy, MPAs were functionalized by attaching alcohol dehydrogenase post-translationally, creating catalytically active magnetic protein aggregates (CatMPAs). These CatMPAs were easily purified from crude extracts via centrifugation or magnetic columns and showed enhanced stability.

**Discussion:**

This study presents a modular strategy for the *in vivo* production of MPAs as scaffold for enzyme immobilization. The approach eliminates the need for traditional, expensive carriers and simplifies the purification process by leveraging the insoluble nature and the magnetic properties of the aggregates, opening up the potential for novel, streamlined applications in biocatalysis.

## 1 Introduction

Enzyme immobilization is a vital technology to obtain reusable and stable biocatalysts with improved properties for industrial application, while remedying the shortcomings of enzymes at the same time; namely, low tolerance to harsh process conditions, stability issues or inhibition of activity (Sheldon and van Pelt, 2013; Datta et al., 2013). To this end, various conventional enzyme immobilization methods exist (Liu, 2020; Mohamad et al., 2015; Homaei et al., 2013), such as physical entrapment where the enzyme of interest is trapped within a membrane or a polymer matrix (Sheldon and van Pelt, 2013), surface immobilization where the enzymes are physically adsorbed onto or covalently linked to the surface of suitable support materials (Barbosa et al., 2015; Bilal et al., 2019), and cross linking (Sheldon, 2011; Jegan Roy and Emilia Abraham, 2004), based on precipitating the proteins from the solution into aggregates (or crystals), followed by cross-linking with a bifunctional reagent. However, these strategies also suffer from various drawbacks such as lowered specific activities, leaching of the enzyme from the support material, high costs associated with carriers and immobilization onto/into such materials, along with labor intensiveness and lack of generalizability (Sheldon and van Pelt, 2013; Wang et al., 2009; Sheldon, 2007; Wahab et al., 2020). Therefore, in recent years, a multitude of alternative, solely biological, *in vivo* enzyme immobilization methods have been developed (Ölçücü et al., 2021; Rehm et al., 2016). These methods, relying on various principles include, amongst others, the display of target proteins on polyhydroxyalkanoate biopolymers generated *in vivo* (Wong et al., 2020; Rasiah and Rehm, 2009), trapping target proteins within biologically produced protein crystals (Heater et al., 2018), generating liquid and hydrogel-like protein condensates based on liquid-liquid phase separation principles (Dzuricky et al., 2020; Wei et al., 2020), or the production of catalytically-active inclusion bodies (CatIBs) (Garcia-Fruitos et al., 2012; Jäger et al., 2020; Diener et al., 2016; Köszagová, 2020). The latter concept requires the fusion of aggregation-inducing peptides/proteins/protein domains to a target protein, resulting in the pull-down of active, correctly folded target within an inclusion body matrix formed by misfolded fusion protein species. All of the aforementioned methods offer numerous benefits from an application point of view, as they do not require the use of additional carrier materials or expensive and time-intensive purification of the target enzyme, and typically yield the desired enzyme immobilizate in one step, directly during heterologous overexpression of the corresponding gene fusions. Therefore, self aggregation/segregation of proteins that partially retain their functionality and can easily be isolated after cell lysis is a highly desired property for potential applications in biotechnology, prompting the need for further developments in the field, e.g., aimed at obtaining magnetic enzyme immobilizates by solely biological means, without the need for *ex vivo* iron loading. To this end, ferritins, a family of ubiquitous, iron-sequestering proteins, which have already been exploited for a wide range of biotechnological applications due to their ability to store iron, high chemical and thermal stability, self-assembling properties and biocompatibility (Arosio et al., 2009; He and Marles-Wright, 2015; Truffi et al., 2016) might represent a promising scaffold. Applications of ferritin include, but are not limited to, serving as a contrast agent for imaging (Wang et al., 2011; Jin et al., 2014), vessel for drug delivery through encapsulation of target molecules (Chiou and Connor, 2018), or in the synthesis of semiconductor nanoparticles (Yamashita et al., 2010). While there are examples of enzyme immobilization where the target enzyme is covalently crosslinked onto (Turan, 2018), or encapsulated within ferritin cages (Bulos et al., 2021; Li et al., 2019), or chemically-loaded magnetoferritin being used for immobilization utilizing the E-coil/K-coil protein-protein interaction (Zhang et al., 2019), the currently available methods either lack general applicability, do not utilize the potential magnetic properties of ferritin, or rely on *ex vivo* iron loading to confer ferritin with magnetism. For instance, encapsulation by ferritin requires either the fusion of another highly positively charged protein to the target, such as GFP(+36), to direct the enzyme into the negatively charged ferritin cavity, or intense genetic modification of the target enzyme to confer it with a highly positive surface charge (Bulos et al., 2021; Li et al., 2019). Further, the small size of the ferritin core allows only limited cargo recruitment (up to 2 enzymes/ferritin cage) and the method does not yield magnetic immobilizates. Similarly, an approach relying on the decoration of ferritin cages with a target enzyme using E-/K-coils requires multiple chromatographic purification steps to obtain E-coil bearing ferritins and K-coil and His-tag bearing target enzyme, where it is necessary for the target to tolerate modifications at both termini (Zhang et al., 2019). Here, additional immobilization of the ferritin-enzyme complex onto an affinity matrix is necessary due to the soluble nature of the enzyme decorated ferritin cages, where the immobilizates reportedly displayed magnetic properties upon *ex vivo* iron loading, despite their aforementioned solubility (Zhang et al., 2019).

Therefore, to the best of our knowledge, there is currently no entirely biological, modular strategy for the generation of magnetic enzyme immobilizates utilizing ferritin, which allows immobilizate purification via magnets without *ex vivo* iron loading. Furthermore, the currently established *in vivo* immobilization methods suffer from the disadvantage of requiring the generation and characterization of numerous constructs consisting of the fusions of the target genes to those of aggregating (Jäger et al., 2020; Ölçücü et al., 2021; Rehm et al., 2016), bio-polymer forming (Grage et al., 2009), or liquid-condensate forming proteins (Schuster et al., 2018), which is a time consuming and laborious process.

To address these shortcomings we developed an *in vivo* immobilization method, based on the self-assembly of ferritin, which offers the following benefits: 1) easy detection of the aggregation efficiency of the scaffold due to the presence of the fluorescent reporter protein, 2) modularity, due to the presence of a protein interaction motif included in the ferritin-based scaffold, which can be easily decorated with soluble target enzymes containing the complementary motif, and 3) two different possibilities to obtain the immobilizates; quickly by simple centrifugation, or with a higher purity via magnetic purification.

**FIGURE 1:**
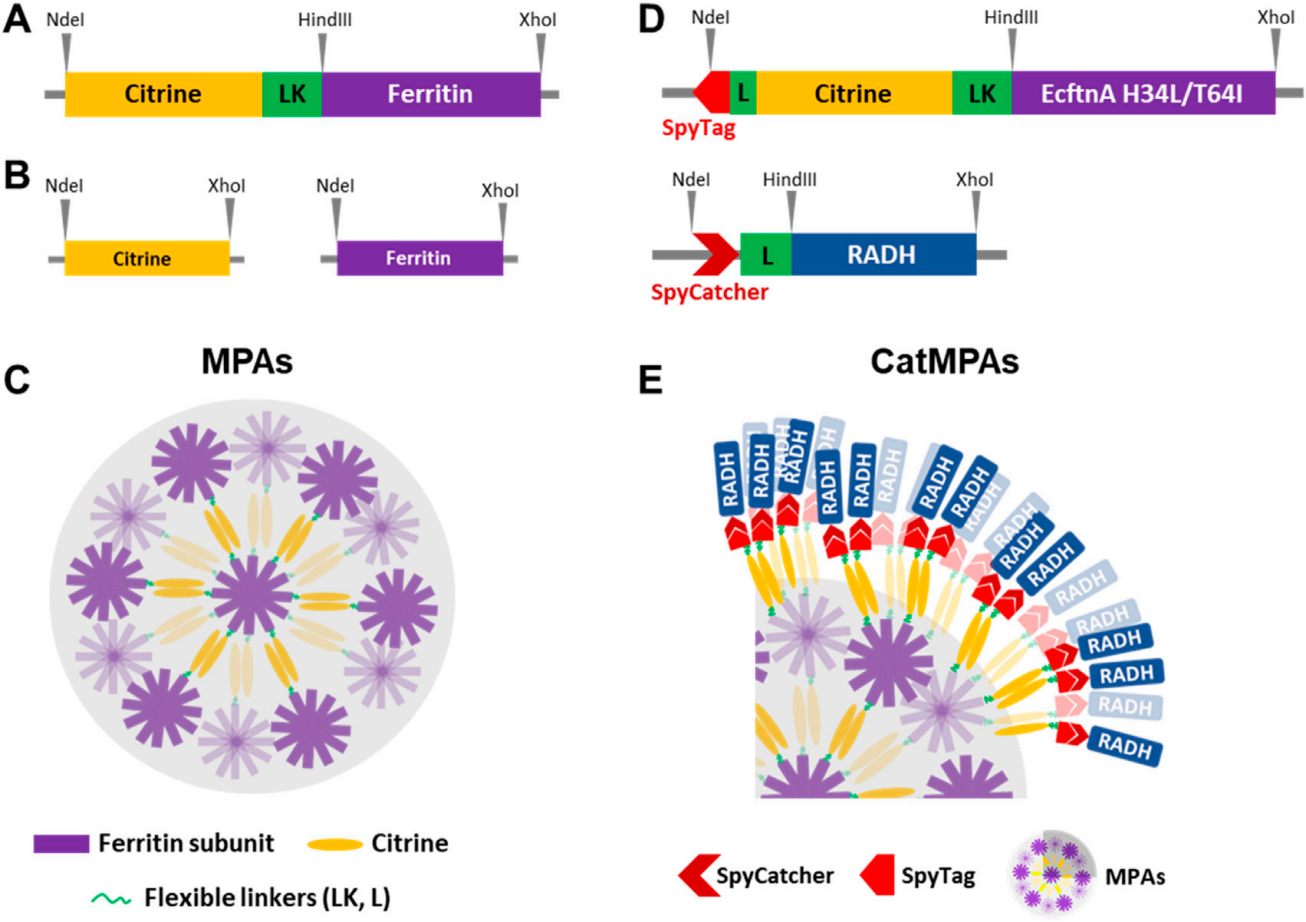
Constructs generated in this study and cartoon diagram depicting the supramolecular assembly of MPAs and CatMPAs. **(A)** Depiction of citrine-ferritin constructs flanked by NdeI and XhoI sites, where a 17-residue linker (LK) with the amino acid sequence GGTGGSGGSGGSGGTGG followed by the HindIII site separates the genes encoding citrine and ferritin. The flexible linker (L) present in CatMPA constructs is similar to the LK linker of MPA, however has the following amino acid sequence: (GGGGS)_2_. Ferritin refers either to the heavy chain of human ferritin (HuftnH), the nonheme ferritin from *E. coli* (EcftnA-WT) or the double mutant of the *E. coli* ferritin (EcftnA H34L/T64I). **(B)** Depiction of the soluble citrine and ferritin constructs flanked by NdeI and XhoI sites. **(C)** Simplified cartoon drawing of the supramolecular assembly of MPAs. Ferritin subunits self-assemble to form the ferritin cage, and the citrines attached to each ferritin subunit form dimers, giving rise to the depicted supramolecular assembly. For simplicity, only half of the ferritin subunits are shown in the cartoon diagram. **(D)** Depiction of the SpyTag/SpyCatcher bearing constructs used in the CatMPA approach. The gene encoding the SpyTag fragment is present at the 5′ of the gene encoding Citrine-EcftnA H34L/T64I fusion in the bait construct, and the gene encoding the SpyCatcher fragment is present at the 5′ of the gene encoding an alcohol dehydrogenase (RADH) for the prey construct. For both SpyTag and SpyCatcher bearing constructs, a flexible (GGGGS)_2_ linker (L) separates SpyTag/SpyCatcher from the remaining gene fusion, whereas the flexible linker (LK) with the amino acid sequence GGTGGSGGSGGSGGTGG is present between citrine and ferritin, similar to MPA constructs. **(E)** CatMPAs produced by mixing the bait and prey fusion proteins depicted in panel **(D)**. The MPAs formed by the SpyTag-Citrine-ferritin (bait) construct are decorated by the SpyCatcher-RADH fusion protein due to the interaction of the SpyTag/SpyCatcher pair. For simplicity of the illustration, only a quarter of the MPA particles depicted in panel **(C)** is shown, and only some of the ferritin subunits are shown to interact with the prey via the citrines, whereas theoretically all 24 ferritin subunits are fused to a citrine bearing the SpyTag which can recruit prey. See methods for additional information and the cloning procedure, and Supplementary Table S1 for the list of all constructs generated in the study.

To this end, we utilized a previously described fusion protein based on the heavy chain of human ferritin (HuftnH) and the yellow fluorescent protein variant citrine that self-assembles into supramolecular complexes *in vivo*, showing sustained self-aggregation and sedimentation upon cell lysis (Bellapadrona and Elbaum, 2014; Bellapadrona et al., 2015). Firstly, we generated and assessed the magnetism of the Citrine-HuftnH fusion first described by Bellapadrona et al. (2015) which did not display substantial magnetic properties in our hands. To implement the envisioned immobilization strategy, we therefore exchanged the HuftnH with various ferritins to generate fully biologically produced, magnetic protein aggregates (MPAs). MPAs containing different ferritins (Figures 1A, C) were characterized with regard to their aggregation efficiencies and magnetic properties, compared to unfused citrine and ferritin control constructs (Figure 1B), and the best performing fusion construct was subsequently used to generate magnetic enzyme immobilizates by using the SpyTag/SpyCatcher protein conjugation system (Zakeri et al., 2012; Zakeri and Howarth, 2010) to allow immobilization of an alcohol dehydrogenase model enzyme, resulting in catalytically-active magnetic protein aggregates (CatMPAs, Figures 1D, E) decorated with the target enzyme. Furthermore, two different approaches to isolate the MPAs directly from the crude cell extracts were developed, which offer the benefit of quick and easy purification via centrifugation, or obtaining high purity via magnetic columns. The ferritin-based CatMPAs therefore represent a modular, novel scaffold to immobilize enzymes, applicable for both *in vivo* and *ex vivo* enzyme immobilization, and thus can serve as a promising new tool for biotechnological applications.

## 2 Materials and methods

### 2.1 Generation of gene fusions and expression constructs

For the generation of Citrine-HuftnH (Bellapadrona et al., 2015), Citrine-EcftnA-WT and Citrine-EcftnA H34L/T64I (Liu et al., 2016) constructs, synthetic genes encoding the fusion proteins flanked by 5′-NdeI and 3′-XhoI sites were synthesized (Invitrogen GeneArt Gene Synthesis, ThermoFischer Scientific). All constructs contained a flexible linker (LK) harboring a 3′-HindIII site between the genes encoding citrine and ferritins. Additionally, since the *ecftna* gene naturally encodes an NdeI site (nucleotides 157–162), the thymine at position 159 was exchanged to cytosine during the design of the genes to simplify the cloning process. Therefore, all EcftnA-WT and EcftnA H34L/T64I constructs generated in this study contained this silent mutation. The plasmids harboring the synthetic genes were hydrolyzed with NdeI and XhoI restriction endonucleases, and were ligated into similarly hydrolyzed pET28a (Merck, Darmstadt, Germany) which was used as expression plasmid. A control strain for the production of soluble citrine lacking ferritin was generated via PCR by employing suitable oligonucleotide primers with 5′-NdeI and 3′-XhoI sites (Supplementary Table S2), using the Citrine-HuftnH construct as template. The resulting PCR product was digested with NdeI and XhoI, and ligated into similarly hydrolyzed pET28a. For the generation of SpyTag002 (Keeble et al., 2017) and SpyCatcher002 (Keeble et al., 2017; Keeble and Howarth, 2019) (optimized variants of SpyTag and SpyCatcher, respectively, referred simply as SpyTag/SpyCatcher in the manuscript) bearing strains, the DNA fragments encoding the SpyCatcher, SpyTag and a (GGGGS)_2_ linker (L) sequence were synthesized (Invitrogen GeneArt Gene Synthesis, ThermoFischer Scientific). The bait construct SpyTag-Citrine-EcftnA H34L/T64I was generated by hydrolyzing the synthetic SpyTag-Citrine gene fusion flanked by 5′-NdeI and 3′-HindIII sites, and ligating the resulting fragment to similarly digested Citrine-EcftnA H34L/T64I containing pET28a vector, and contained the linker (L) sequence separating the gene encoding SpyTag from the gene fusion encoding citrine-ferritin. The prey construct SpyCatcher-RADH was generated by the amplification of the synthetic SpyCatcher sequence using primers with 5′-NdeI and 3′-HindIII sites (Supplementary Table S2), followed by hydrolyzing the PCR product by these restriction enzymes, and ligating it to similarly hydrolyzed vector containing the RADH sequence that was generated elsewhere (Ölçücü et al., 2022).The SpyCatcher-RADH construct hence contained a cleavage site for the Factor Xa protease followed by a HindIII site at the 3′ end of the linker (L) separating the genes encoding SpyCatcher and RADH). All constructs were verified by sequencing (Seqlab GmbH, Göttingen, Germany).

### 2.2 Bacterial strains, media and cultivation

*E. coli* DH5α served as the cloning host for the generation of the constructs. For heterologous expression, *E. coli* BL21 (DE3) was used. Lysogeny broth (Bertani, 1951) served as the growth medium for the cultivation, during cloning and for the precultures for heterologous overexpression of the gene fusions. Autoinduction (AI) medium (Studier, 2005) (12 g/L casein-hydrolysate, 24 g/L yeast extract, 2.2 g/L KH_2_PO_4_, 9.4 g/L K_2_HPO_4_, 5 g/L glycerol at pH 7.2 supplemented with 0.5 g/L glucose and 2 g/L lactose) was used as the growth medium during protein production. 50 μg/mL kanamycin was added to all growth media for plasmid maintenance. Briefly, LB precultures were used to inoculate the expression cultures with an initial OD_600_ of 0.05 and were cultivated at 37°C for 3 h, shaking at 130 rpm. After 3 h, the expression cultures were supplemented with iron-citrate complex to a final concentration of 1 mM (0–10 mM iron-citrate for BioLector experiments with varying iron concentrations, Supplementary Figure S2), using a sterile filtered stock solution of 100 mM FeSO_4_·7H_2_O-500 mM citrate pH 7. Subsequently, all cultures were transferred to 15°C and incubated for another 69 h at 130 rpm. For microscopy, soluble citrine and Citrine-HuftnH/EcftnA-WT/EcftnA H34L/T64I producing strains were cultivated in a BioLector setup in M9-AI medium (5 g/L (NH4)_2_SO_4_, 3 g/L K_2_HPO_4_, 6.8 g/L Na_2_HPO_4_, 0.5 g/L NaCl, 2 g/L NH_4_Cl, 0.2 g/L MgSO_4_·7H_2_O, 1.5 mg/L CaCl_2_· 5H_2_O, 15 mg/L FeSO_4_, 0.2 g/L Na_3_C_6_H_5_O_7_·2H_2_O, 10 mg/L thiamine, 0.75 mg/L AlCl_3_·6H_2_O, 0.6 mg/L CoCl_2_·6H_2_O, 2.5 mg/L CuSO_4_·5H_2_O, 0.5 mg/L H_3_Bo_3_, 17.1 mg/L MnSO_4_·H_2_O, 3 mg/L Na_2_MoO_4_·2H_2_O, 1.7 mg/L NiCl_2_·6H_2_O, 15 mg/L ZnSO_4_·7H_2_O, 5 g/L glycerol, 0.5 g/L glucose and 2 g/L lactose) supplemented with 1 mM iron-citrate and were inoculated to a starting OD_600_ of 0.05 from LB precultures grown overnight. The initial cultivation was performed at 37°C for 3 h shaking at 1,200 rpm, and expression took place at 15°C for 69 h at 1,200 rpm, after which the live cells were imaged (Section 2.8).

### 2.3 Preparation of cell fractions

*E. coli* BL21 (DE3) cells overproducing the target proteins or protein fusions were harvested by centrifugation (6500 × g, 30 min, 4°C). Cells were resuspended to 10% (w/v) in lysis buffer (50 mM sodium phosphate buffer, 100 mM NaCl, pH 7.0 for SpyTag/SpyCatcher bearing CatMPA constructs, pH 8.0 for the remaining constructs). For CatMPA constructs, the lysis buffer at pH 7.0 also served as the incubation buffer for the SpyTag-SpyCatcher reaction to take place. Cells were lysed by using an Emulsiflex-C5 high pressure homogenizer (Avestin Europe GmbH, Mannheim, Germany) with internal pressure between 1,000 and 1,500 bar, 3 cycles under constant cooling. For CatMPA constructs, the freshly obtained crude cell extracts (CCEs) of bait and prey were mixed in 1:1 (v/v) ratio, vortexed for a few seconds, and then incubated at 25°C for 30 min with shaking at 600 rpm. After 30 min, the mixed CCEs were placed on ice. To obtain the soluble and insoluble cell fractions, fresh CCE (or the CCE mixture of bait + prey) was diluted using lysis buffer, and half of the diluted CCE was centrifuged for fractionation (7697 × *g*, 2 min, room temperature) as described elsewhere (Ölçücü et al., 2022). The supernatant (S) was transferred to a fresh tube and the unwashed pellet (P1) was resuspended using the same volume of lysis buffer as the removed S fraction. The suspended pellet was centrifuged (7697 × *g*, 2 min, room temperature), and the supernatant of the wash (S2) was discarded. The washed pellet was resuspended again in the same volume of lysis buffer as the removed supernatant, resulting in the washed pellet fractions (P). The obtained cell fractions (CCE, S and P) were subsequently kept on ice and were used to determine the fluorescence/RADH activity distributions of the constructs and their mixtures. The MPA pellets were further lyophilized as described elsewhere [(Ölçücü et al., 2022), see also SI methods] to determine yields, and the respective CatMPA and bait-prey pellets were lyophilized similarly for stability analyses.

### 2.4 Imaging over permanent neodymium magnets

To visualize the magnetic properties of citrine-ferritin fusions, 5 mL of crude cell extracts (CCE) of constructs overproducing the citrine-ferritin fusions were mixed with 1 mL of OptiPrep Density Gradient Medium (STEMCELL Technologies Germany GmbH, Köln, Germany) and transferred to mini petri dishes, corresponding to 10% iodixanol (w/v) concentration in the mixture. The CCE-OptiPrep mixture was supplemented with 50 μg/mL kanamycin to prevent contamination during the course of imaging. Four permanent, axially magnetized N45 neodymium ring magnets (with the dimensions of 20 mm (outer diameter), 10 mm (inner diameter), 6 mm height, EarthMag GmbH, Dortmund, Germany) were arranged in a 2 × 2 grid and were used to assess the magnetic properties of the constructs visually. Black papers, cut in a rectangular shape, were placed over the neodymium magnets to aid visualization in a similar way as described elsewhere (Liu et al., 2016; Nishida and Silver, 2012), and the mini petri dishes containing the CCEs were placed carefully on top of the papers resting over the neodymium magnets. The samples were imaged every 10 min for up to 69 h, and the patterns emerging in the solution due to the attraction of the citrine-ferritin particles towards the neodymium magnets were captured using a camera (Logitech C930E Full HD-Webcam, Logitech Europe S.A., Lausanne, Switzerland) which was placed directly above the samples. Time lapse videos were created using images taken at 10-min intervals via SkyStudioPro, and edited using DaVinci Resolve 17 (Blackmagic Design Pty Ltd., Melbourne, Australia) to minimize flickering.

### 2.5 Magnetic column purification

To magnetically purify ferritin fusion proteins, commercial MS columns containing ferromagnetic spheres were placed in an OctoMACS separator containing a permanent magnet, held by a MACS MultiStand (Miltenyi Biotec B.V. & Co. KG, Bergisch Gladbach, Germany). The crude cell extracts of MPAs and CatMPAs were supplemented with 0.05 mg/mL DNase I to prevent clogging of the MS columns prior to application. The lysis buffer (50 mM sodium phosphate buffer, 100 mM NaCl, pH 7.0 for SpyTag/SpyCatcher constructs and pH 8.0 for the remaining constructs) was degassed to get rid of air bubbles that could likewise clog the column. 1 mL of degassed lysis buffer was used to wet the MS column placed in an OctoMACS separator and the eluate was discarded. After the equilibration step, 1 mL of CCE was passed through the MS column and collected, and the eluted CCE sample was reloaded onto the same MS column for a total of three times. The sample that eluted after the third run was collected and labelled as the NM (nonmagnetic) fraction. The column was then washed two times using 1 mL degassed lysis buffer and the eluates were collected separately as wash fractions W1 and W2. To obtain the MG (magnetic) fraction, the MS column was removed from the OctoMACS separator, loaded with 1 mL degassed lysis buffer, and the magnetic particles suspended in the column were quickly flushed out using the plunger provided in the kit and collected in a separate tube. All fractions were kept on ice until further analysis.

### 2.6 Fluorescence spectrophotometry

Fluorescence emission of cell fractions of the citrine-containing fusions were measured in quadruples using black Nunc 96-Well MicroWell polypropylene plates (ThermoFisher Nunc, Waltham, United States) and a TECAN infinite M1000 PRO fluorescence MTP reader (TECAN, Männedorf, Switzerland). 100 μL of CCE, S, P cell fractions or NM, W1, W2, MG fractions in appropriate dilutions were applied in quadruples onto the microtiter plates and the fluorescence emission of the samples were quantified (λ_ex_ = 513 nm, λ_em_ = 529 nm, z-position 18.909 µm, enhancement 120, flash number 25, flash frequency 400 Hz, bandwidth 5 nm). Samples were shaken (654 rpm, 2 mm amplitude) for 5–10 s immediately before the fluorescence measurements to ensure that the particles are suspended. All measurements were performed using at least three biological replicates.

### 2.7 RADH activity measurements

The cell fractions of prey constructs containing RADH, along with the respective fractions of CatMPA CCE mixtures were tested for the distribution of the RADH activity using a discontinuous photometric assay where the consumption of the NADPH was detected as described earlier (Jäger et al., 2019; Ölçücü et al., 2022). Briefly, RADH containing cell/magnetic purification fractions and a reaction mixture of 1,400 µL containing 0.5 mM NADPH and 125 mM cyclohexanone in TEA-buffer (50 mM Triethanolamine, 0.8 mM CaCl_2_, pH 7.5) were incubated separately at 30°C for 5 min. The reaction was initiated by transferring 350 µL of the RADH containing sample onto the 1,400 µL reaction mixture, immediately vortexed, and a sample of 250 µL was taken which was transferred onto 500 µL of methanol to stop the reaction. The remaining reaction mixture was quickly placed in a shaking incubator at 1,000 rpm and 30°C. The rest of the reaction mixture was then sampled every minute for a total of six times in the same manner, where the sampled reaction was stopped in methanol. After the last sampling step, the vials were centrifuged (7697*g*, 5 min, room temperature) and transferred to disposable cuvettes to measure the absorption spectra (280–500 nm) using a Cary 60 UV-Vis Spectrophotometer (Agilent, Santa Clara, United States). All measurements were performed using at least three biological replicates. Stability analyses were performed as described previously (Ölçücü et al., 2022). In brief, 14 mg of CatMPA or the SpyCatcher-RADH prey lyophilizates were suspended in 7 mL of lysis buffer (50 mM sodium phosphate buffer, 100 mM NaCl, pH 8.0) and vortexed until no visible clumps remained in the solution. The RADH activity of the lyophilizate suspensions were measured in triplicates as described above for the cell fractions, where the reaction mixture was sampled once every 2 min instead of 1 min, for a total of 10-min assay time, in order to provide increased sensitivity. After the assay, the remaining lyophilizate suspensions were placed at a 25°C incubator for 5 days, where the RADH activity assay was repeated in triplicates every 24 h.

### 2.8 Microscopic analyses

Live *E. coli* BL21 (DE3) cells producing the MPA constructs and soluble citrine were cultivated in M9-AI medium supplemented with 1 mM iron-citrate complex as described above. At the end of expression (69 h), cultures were diluted appropriately in lysis buffer (50 mM sodium phosphate buffer, 100 mM NaCl, pH 8.0) and 1 µL of the cell suspension was transferred to glass slides and covered with a coverslip. The samples were then analyzed with a Nikon Eclipse Ti microscope (Nikon GmbH, Düsseldorf, Germany) equipped with a Nikon DS-Qi2 camera (Nikon GmbH, Düsseldorf, Germany) and a YFP filter (λ_ex_ = 500 nm, λ_em_ = 542.5 nm). Fluorescence and camera exposure times were 200 ms for ph3 and 100 ms for the YFP filter used to detect citrine fluorescence. For CatMPA constructs, approximately 1 mL was sampled at the end of expression, centrifuged (7697 g, room temperature, 1 min), resuspended and diluted suitably using lysis buffer. The cell suspension was then transferred to polydimethylsiloxane microfluidic chips with inner chamber dimensions of 60 μm × 100 μm × 1 µm, and imaged using a Nikon Eclipse Ti microscope (Nikon GmbH, Düsseldorf, Germany) with a YFP filter block (λ_ex_ 495 nm, λ_em_ 520 nm) and Andor Zyla VSC-01418 camera (Oxford Instruments plc, Oxon, United Kingdom) with exposure times of 100 ms for ph3 and YFP filters. Samples for scanning electron microscopy analyses were prepared by dissolving the 1 mg of lyophilized MPA/CatMPAs in 50 mM sodium phosphate buffer pH 7.0, supplemented with 100 mM NaCl. Samples were applied to poly-l-lysine coated cover glasses and fixed with 4% glutaraldehyde in 50 mM sodium phosphate buffer pH 7.0, supplemented with 100 mM NaCl by incubation for 2 h at room temperature. Subsequently, the samples were washed three times, 10 min each, with 50 mM sodium phosphate buffer pH 7.0, supplemented with 100 mM NaCl, stored overnight at 4°C and dehydrated by using a graded ethanol series (30%, 50%, 70%, 95%, and 100%), with each step lasting 15 min. Finally, samples were dried by critical point method and sputter-coated with gold. Scanning electron microscopy images of lyophilized MPAs and CatMPAs were taken by Miriam Bäumers from the Center for Advanced Imaging (CAi) at the Heinrich-Heine University Düsseldorf with a Zeiss Supra 55VP scanning electron microscope (Carl Zeiss AG, Oberkochen, Germany) equipped with an angle-selective back-scattering detector (AsB). Images were taken at an accelerating voltage of 10 kV.

### 2.9 Determination of protein concentration and SDS-PAGE analyses

Protein concentration of the supernatant samples (S) were determined with the Bradford assay (Bradford, 1976) and bovine serum albumin standards with concentrations between 0.01–0.1 mg/mL. Either home-made SDS-gels (5%–12%) or NuPAGE 4%–12% Bis-Tris protein gels in MES SDS running buffer (50 mM MES, 50 mM Tris, 0.1% SDS, 1 mM EDTA, pH 7.3) were used for SDS-PAGE analyses. The volume required to have 10 µg of protein based on the Bradford assay for the S fraction was set as the loading volume for the remaining cell and magnetic purification fractions except for MG. For the MG fraction, the sample was applied onto polyethersulfone membrane centrifugal filters with 3 kDa cutoff (VWR International GmbH, Darmstadt, Germany) to concentrate this fraction. The concentrated MG sample was loaded onto the SDS gel to contain 20 µg of protein per lane in order to increase the sensitivity to detect impurities. Cell fractions were boiled for 3 min at 100°C before loading onto the SDS gels, and each gel contained 3 µL PageRuler Prestained Protein Ladder (ThermoFisher Nunc, Waltham, United States).

## 3 Results and discussion

### 3.1 Diversification of a ferritin-based self-assembly system

**FIGURE 2:**
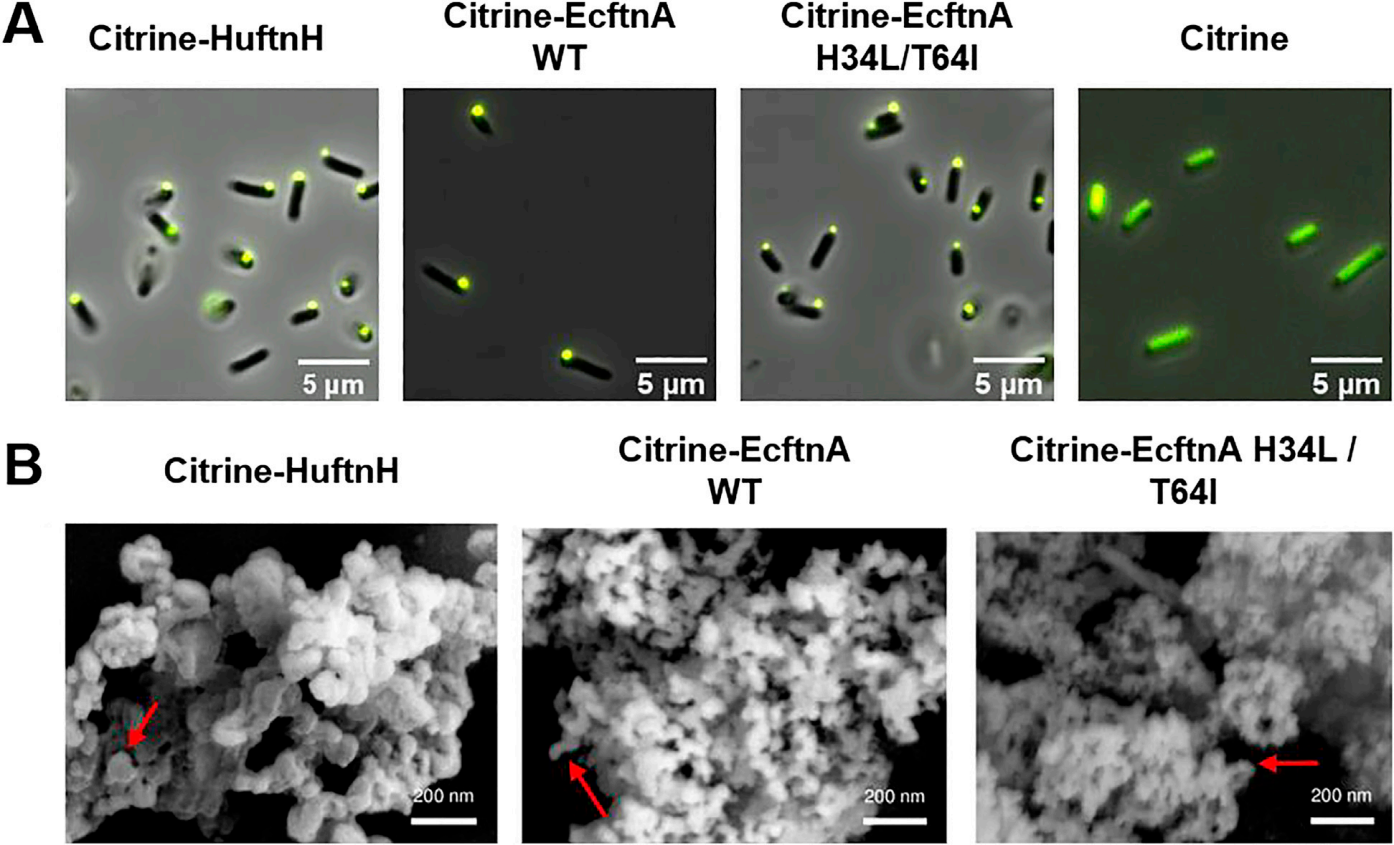
Microscopic analyses conducted on MPAs and soluble citrine. **(A)** Fluorescence microscopy pictures of live *E. coli* BL21 (DE3) cells overproducing citrine-ferritin fusions and soluble citrine. **(B)** Scanning electron microscopy images of lyophilized MPAs. See methods section for details and cultivation conditions. The red arrows indicate the smoother, spherical regions which make up the larger MPA aggregate.

**FIGURE 3:**
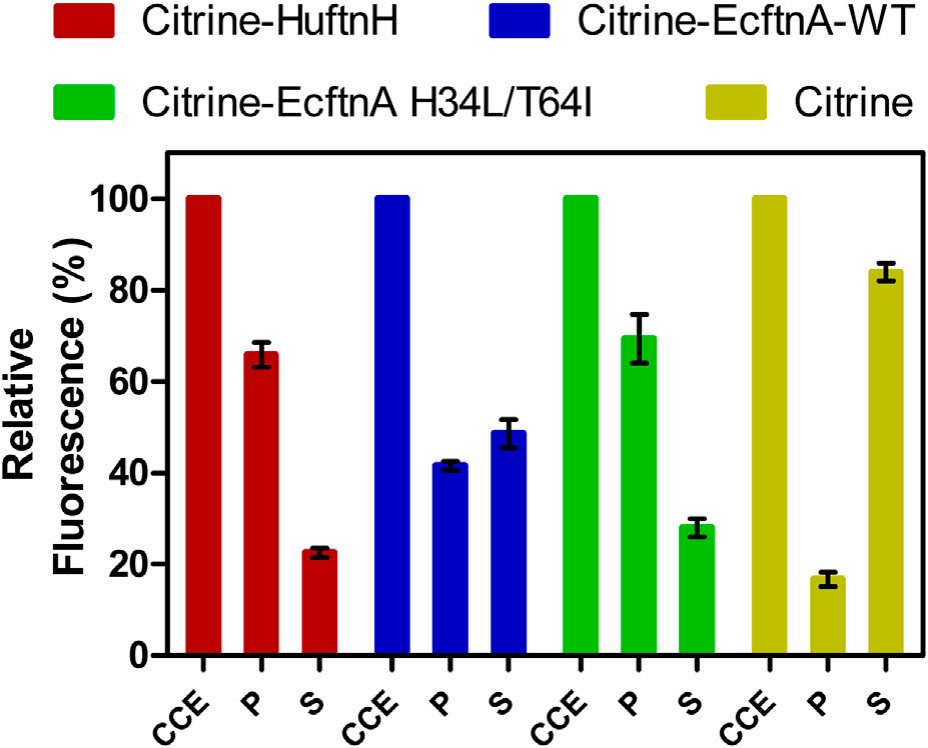
Relative fluorescence of cell fractions of citrine-ferritin fusions and soluble citrine producing cells. Citrine fluorescence of the crude cell extract (CCE) fraction was set to 100% for each construct, and the fluorescence signal detected in the washed pellet (P) and supernatant (S) fractions is shown relative to the fluorescence of their corresponding CCE fractions. The error bars represent the standard error of the mean derived from at least three biological replicates with four technical replicates each.

To obtain biologically produced, magnetic immobilizates, we initially reconstructed a fusion protein consisting of the fluorescence reporter citrine and the heavy chain of human ferritin (Citrine-HuftnH) as first described by Bellapadrona and co-workers (Bellapadrona et al., 2015; Bellapadrona and Elbaum, 2014). Citrine-HuftnH had been shown to yield self-assembling supramolecular complexes, producing fluorescent particles in *E. coli*, which further aggregated and sedimented in solution upon release from the cells. Self-assembly and aggregation was postulated to be due to dimerization of citrine attached to the ferritin subunits that themselves assemble to intact ferritin cages, with the citrines mediating the formation of the supramolecular complexes (Bellapadrona and Elbaum, 2014; Bellapadrona et al., 2015) (Figure 1C). To extend on this strategy, we used a nonheme *E. coli* ferritin (EcftnA-WT), and a magnetically enhanced EcftnA H34L/T64I variant (Liu et al., 2016) in addition to HuftnH, and fused the genes encoding the human and *E. coli* ferritins to the 3′ end of the gene encoding citrine (Figure 1A). For initial assessment of the self-aggregation properties of all constructs, Citrine-HuftnH, Citrine-EcftnA-WT and Citrine-EcftnA H34L/T64I fusions were overproduced in *E. coli* BL21 (DE3) and the cells were lysed to yield the crude cell extract (CCE) fractions. All CCEs visually showed self-aggregation and sedimentation when left undisturbed (Supplementary Figure S1), confirming that the exchange of human ferritin with *E. coli* ferritins did not interfere with the aggregation tendency of the fusion proteins. The presence of intracellular supramolecular aggregates was further confirmed via microscopic analyses conducted on live *E. coli* BL21 (DE3) cells overproducing the citrine-ferritin fusions, with a construct producing soluble citrine included as a negative control (Figure 2A). All citrine-ferritin fusions exhibited localized signals for citrine fluorescence at only one of the cell poles, whereas citrine control construct displayed uniform fluorescence as expected. This observation is also in line with the relative fluorescence data (Figure 3), and the literature on the Citrine-HuftnH construct (Bellapadrona et al., 2015), where Citrine-HuftnH exhibited localized fluorescence signals. It should be noted that the aggregates produced by citrine-ferritins are visually different when compared to conventional (catalytically-active) inclusion bodies (CatIBs) (Jäger et al., 2019; Jäger et al., 2018; Garcia-Fruitos et al., 2005; Ölçücü et al., 2022), as the citrine-ferritin aggregates appear smaller in size and are predominantly present at just 1 cell pole, as opposed to inclusion bodies which are in general present at both poles. However, the number of generations after induction can have a significant impact on the number and size of the inclusion bodies (IBs) found in bacteria. For instance, spherical IBs are more commonly observed in ‘older’ cell cultures, and once IB size is sufficiently large, they tend to fuse into one larger IB, which is thought to occur via the agglomeration of the rod-shaped, early-stage IBs (Kopp et al., 2023).

While the time between induction and harvest is sufficient for the formation of a single, spherical IB in the MPA producing cells in our experiments, the smaller size of the fluorescent signal originating from the single pole in comparison to CatIBs which were imaged under the same conditions (Jäger et al., 2019; Jäger et al., 2018; Garcia-Fruitos et al., 2005; Ölçücü et al., 2022), as well as the lack of refractile particles under phase contrast (data not shown) which is a typical feature of IBs/CatIBs, indicate that MPAs are likely not IB-based materials. This is further corroborated by preliminary scanning electron microscopy studies (Figure 2B) carried out to reveal the overall morphology of MPAs. As can be seen from these analyses, MPAs are rather heterogeneous agglomerates, whose aggregation is likely driven by the dimerization of citrines present on the surface of the ferritin cages (see Figure 1C). The detected aggregates contained spherical regions with a somewhat smooth appearance (Figure 2B, marked by the red arrows), approximately 50–100 nm in diameter, which likely represent larger aggregates of multiple ferritin cages. These spherical regions appear to make up the larger clusters with diverse 3D structures, which consist of branched and clumped areas, up to several micrometers in size. While the individual ferritin cages (with a diameter of approx. 12 nm) are too small to be visible in SEM images, the MPAs formed by the fusion of citrine to HuftnH appeared to contain somewhat larger spherical regions (≈100 nm) that make up the larger aggregates, compared to those formed by the fusion of citrine to *E. coli* ferritins (≈50 nm). Aggregates as small as 150 nm, which correspond to a cluster of 2-3 spherical regions, as well as much larger aggregates, whose length exceeded 6 µm could be detected, with no clear correlation between the type of ferritin used and the size of the larger aggregates. As the size of the largest MPAs exceed the size of an *E. coli* cell, it is likely that the largest aggregates form after cell lysis, which is in line with the smaller size of the MPAs observed via the fluorescence microscopy analyses conducted on live cells (Figure 2A). Furthermore, the single, spherical, fluorescent spots seen at the cell poles of MPA-producing *E. coli* cells (Figure 2A), likely represent self-assembled MPAs particles and not individual cages, which would be too small to detect. This is in contrast to SEM analyses of CatIBs which form rod-shaped or spherical particles with a size of 50–1,000 nm, with individual CatIB particles being detectable as refractile particles at both cell poles by phase contrast and fluorescence microscopy (Gil-Garcia and Ventura, 2021). These observations collectively stress the question about the nature of the MPA aggregation process *in vivo*. Given our SEM analyses, it seems unlikely that MPAs simply represent magnetized CatIBs of ferritin, but rather form by different means. Possible processes include liquid-liquid-phase separation (LLPS), which is known to occur for human ferritin, in the physiological context, driven by multivalent interactions with the nuclear receptor coactivator 4 (NCOA4) protein (Ohshima et al., 2022). To address this possibility, however, further studies utilizing purified Citrine-HuftnH, Citrine-EcftnA-WT and Citrine-EcftnA H34L/T64I fusions would be needed, which are beyond the scope of the present contribution.

To quantify the aggregation efficiencies of all constructs, CCEs were fractionated (see Preparation of cell fractions) by centrifugation to yield the soluble supernatant (S) and the insoluble pellet fractions for all constructs. The pellets were then washed and centrifuged a second time to yield the washed pellet (P) fractions, which allowed the quantification of citrine fluorescence distributions for all constructs (Figure 3). Citrine fluorescence detected in the P fraction was then compared to the fluorescence of the CCE fraction (set to 100%) to assess the aggregation efficiencies for all constructs. A construct overproducing soluble citrine was also included in the analysis as control. As evident from Figure 3, all pellets obtained from the citrine-ferritin fusions were fluorescent and the Citrine-EcftnA H34L/T64 construct displayed the highest aggregation efficiency among the generated constructs, where 69% of the total fluorescence signal of the CCE originated from the insoluble, washed pellet fraction for this construct. Citrine-HuftnH and Citrine-EcftnA-WT constructs displayed similarly high efficiencies (66% and 42%, respectively). In contrast, the citrine construct lacking ferritin showed only 17% of the citrine fluorescence in the pellet, indicating that in addition to dimerization of citrines, fusion of ferritin to the citrine is crucial for aggregation, which is in line with earlier studies conducted with the HuftnH fusion construct (Bellapadrona et al., 2015; Bellapadrona and Elbaum, 2014). In addition, yields of the constructs were determined, along with their protein contents (Supplementary Table S4), which indicates that the ferritin-based protein aggregates can be produced at comparable yields (up to 4.7 g lyophilizate/100 g wet cells, and up to 77% protein content depending on construct, see Supplementary Table S4 and SI for the method) as compared to CatIBs (Ölçücü et al., 2022).

In conclusion, we could demonstrate that the HuftnH can be successfully exchanged with *E. coli* ferritins to obtain fluorescent aggregates, and, as evidenced by the case of the EcftnA H34L/T64I mutant, the resulting fusion proteins can exhibit superior aggregation efficiencies.

### 3.2 Magnetic properties of MPAs generated by citrine-EcftnA H34L/T64I and magnetic purification

**FIGURE 4:**
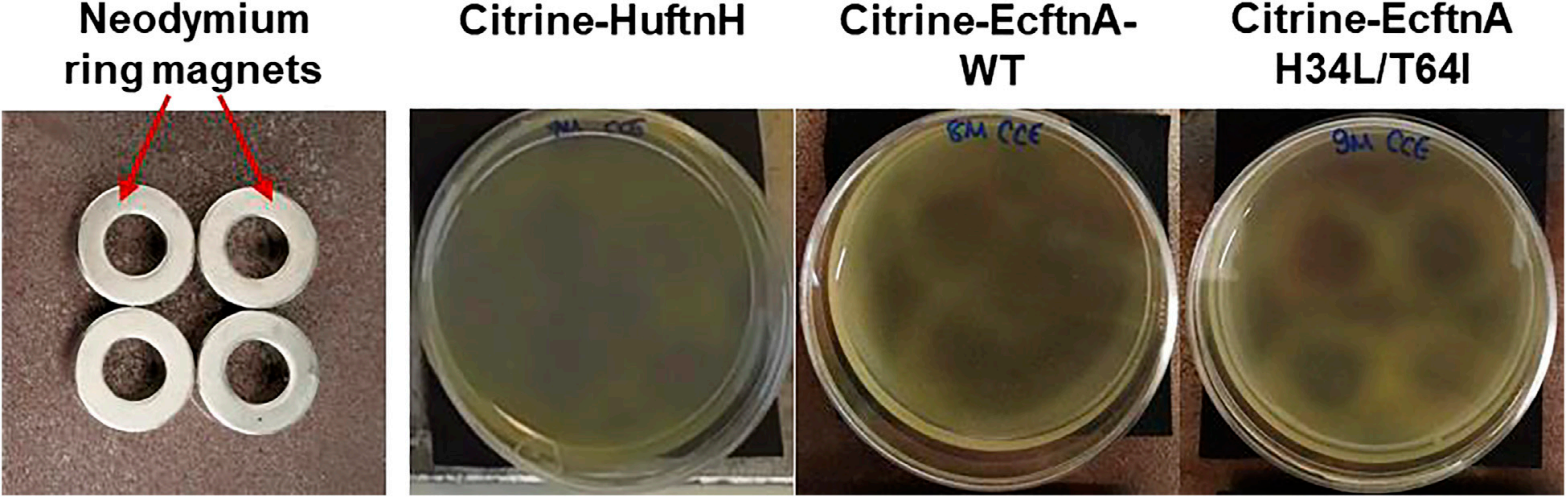
Assessment of the magnetic properties displayed by the crude cell extracts (CCEs) containing different citrine-ferritin fusion proteins using permanent neodymium ring magnets. The magnets were arranged in a 2×2 grid as shown in the leftmost panel, and covered with a black paper to aid visualization, onto which the mini petri dishes containing the CCE-OptiPrep density gradient medium mixtures (17% OptiPrep) were placed. Upon placement over the permanent magnets, the CCEs were left undisturbed for up to 69 h to follow the pattern formation. The contrast of all images was increased by 20%.

After the initial characterization of citrine-ferritin fusions via live cell microscopy, SEM and fluorescence spectroscopy, we investigated the magnetism of citrine-ferritin fusions. To provide an easy, visual indication of the magnetic properties of Citrine-HuftnH, Citrine-EcftnA-WT and Citrine-EcftnA H34L/T64I fusions, the fluorescent citrine-ferritin particles were tested for their response towards permanent magnets, in a similar way that was described elsewhere to test whole cell magnetism (Nishida and Silver, 2012; Liu et al., 2016). To this end, cells overproducing the citrine-ferritin fusions, which were cultivated in autoinduction medium supplemented with 1 mM iron-citrate complex, were lysed (see methods for details and Supplementary Figure S2 for BioLector experiment with varying iron concentrations). The crude cell extracts (CCEs) of the citrine-ferritin fusions were then transferred to mini petri dishes containing 17% (v/v) OptiPrep density gradient medium. CCE-OptiPrep suspensions were immediately placed over permanent neodymium ring magnets (arrangement shown in Figure 4, leftmost panel) and were imaged up to 69 h using a camera placed above the samples.

The attraction of citrine-ferritin particles in the CCE towards the neodymium magnets underneath the suspensions gave rise to patterns of varying intensity for the tested constructs (Figure 4). Faint, albeit noticeable patterns started forming as early as 6 hours for the CCE of Citrine-EcftnA H34L/T64I construct, and after 12 h, faint patterns were visible for all three citrine-ferritin constructs (SI video, see Supplementary Figure S4 for images of citrine-ferritin CCEs visualized every 6 h). Imaging was continued for a total of 69 h to ensure capturing of the entire pattern progression, which became noticeably sharper for the Citrine-EcftnA H34L/T64I construct as time progressed. As a negative control, cells overproducing HuftnH, EcftnA-WT and EcftnA H34L/T64I without fused citrine were cultivated and lysed under identical conditions, and their CCEs were placed over permanent magnets as well, which showed no distinct pattern formation (Supplementary Figure S3), indicating that the soluble ferritins that lack citrine do not form substantial (magnetic) aggregates.

**FIGURE 5:**
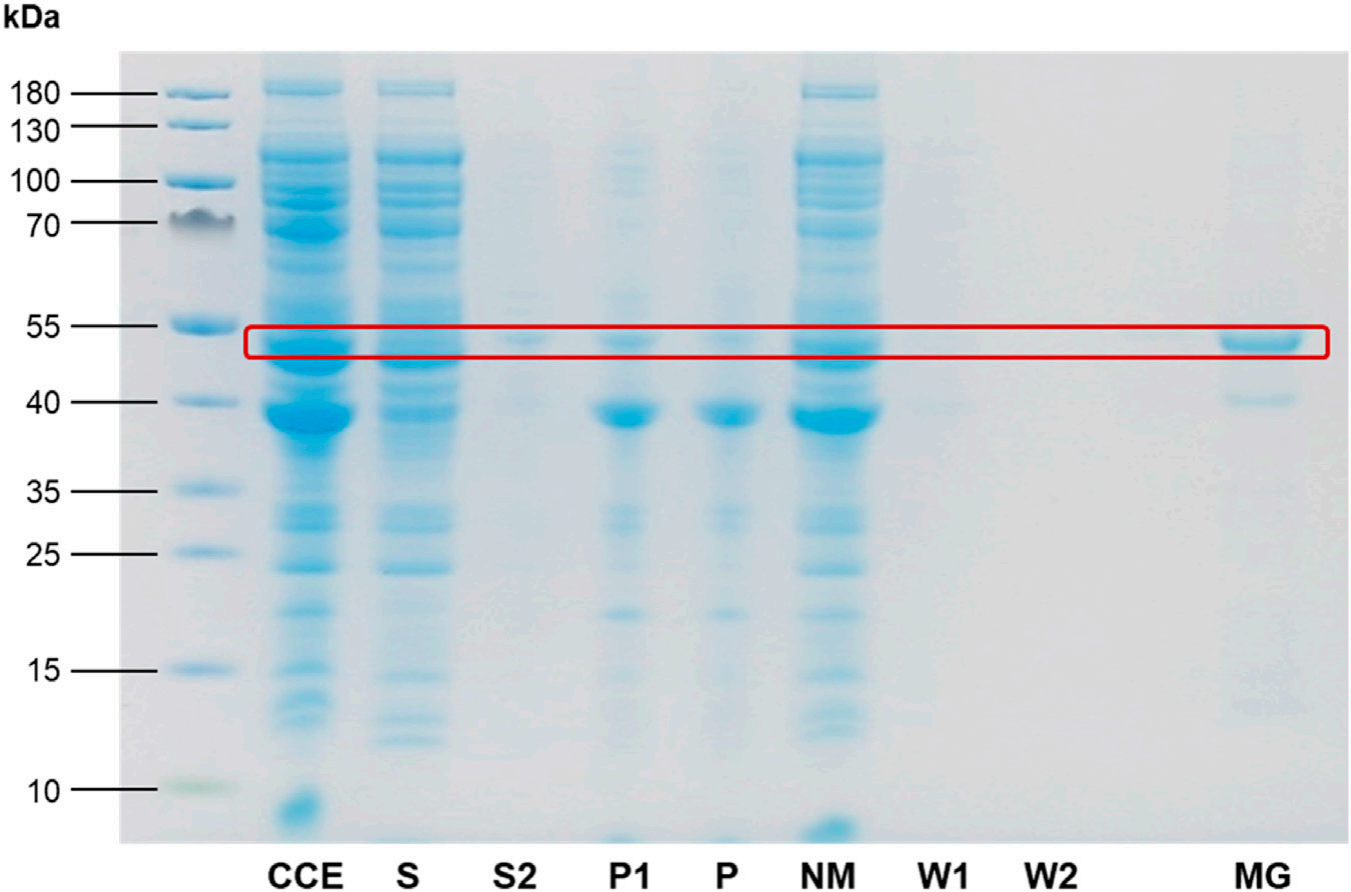
SDS-PAGE analysis of Citrine-EcftnA H34L/T64I cell fractions and the magnetic purification process. The expected molecular weight of the Citrine-EcftnA H34L/T64I fusion protein (47.8 kDa) is marked with a red rectangle for all fractions. CCE: crude cell extract, S (1): supernatant, S2: supernatant of wash step, P1: unwashed pellet, P (2): washed pellet, NM: nonmagnetic fraction, W1: first wash, W2: second wash, MG: magnetic fraction. Protein content of the S fraction was determined using the Bradford assay, and the volume required to load 10 µg protein for the S fraction was used as the sample volume for all remaining fractions except for MG fraction. The concentration of the MG fraction was determined separately, and the fraction was concentrated prior to loading in order to contain 20 µg protein to assess purity of the fraction more critically (See Methods for details, and Supplementary Figure S5 for SDS-PAGE analyses of cell fractions from all soluble control, MPA, and CatMPA constructs).

In conclusion, imaging CCEs of the citrine-ferritin fusions over permanent magnets provided first insights into the magnetic properties of the corresponding MPAs, where the Citrine-EcftnA H34L/T64I construct surpassed Citrine-HuftnH and Citrine-EcftnA-WT constructs in this regard. In addition to showing superior magnetic properties, the Citrine-EcftnA H34L/T64I construct also showed the highest aggregation efficiency as judged by the fluorescence distribution data (Figure 3). Therefore, all further work was conducted using this construct. The magnetic properties of the Citrine-EcftnA H34L/T64I MPAs were further exploited to purify the fusion protein using MS magnetic columns and OctoMACS separator system (Miltenyi Biotec). In brief, the CCE of Citrine-EcftnA H34L/T64I was passed through the same MS column for a total of three times and the eluate was collected (nonmagnetic fraction, NM). The column was then washed twice using lysis buffer (50 mM sodium phosphate buffer, 100 mM NaCl, pH 7.0) and the wash fractions (W1 and W2) were collected. Finally, the magnetic (MG) fraction was eluted by separating the column from the OctoMACS permanent magnet, applying lysis buffer onto the column and quickly flushing the MG fraction using a small plunger. The magnetic column purification fractions were then loaded onto an SDS-PAGE along with the cell fractions obtained via centrifugation, for the assessment of purity of the Citrine-EcftnA H34L/T64I protein. SDS-PAGE analysis revealed that the Citrine-EcftnA H34L/T64I fusion protein can be purified using magnetic columns, evident by the clear band present in the MG fraction (Figure 5). Furthermore, the washed pellet fraction (P) of the centrifugation approach containing MPAs contained other proteins as well (i.e., possibly chaperons and membrane proteins commonly encountered in CatIB approach (Kloss et al., 2018) for such insoluble fractions), whereas the magnetically purified MPAs were of high purity. Subsequently, the wash fractions of the magnetic purification samples (W1 and W2) were clear, indicating that the columns retain the Citrine-EcftnA H34L/T64I fusion protein rather well, therefore, using magnetic columns appears as a suitable method for purifying MPAs. Furthermore, the citrine-specific fluorescence of the fractions obtained from the magnetic purification approach were determined, and fluorescence detected in each fraction was compared to the total fluorescence of the CCE (set to 100%). Unfortunately, the majority of the citrine fluorescence (approximately 80% of the total CCE fluorescence) originated from the nonmagnetic (NM) fraction, followed by 19% for the magnetic (MG) fraction. The wash fractions W1 and W2 displayed almost no fluorescence (4% and 0.3% when compared to CCE, respectively). As the majority of the fluorescence detected for the Citrine-EcftnA H34L/T64I MPA construct originated from the insoluble fraction (Figure 3), this result indicates that not all of the citrine-ferritin aggregates could be purified by the magnetic purification approach. This could be due to several factors: 1) a significant fraction of citrine-ferritin aggregates exhibits weaker magnetism and are not retained by the column (i.e., due to unequal loading of individual ferritin cages); 2) the majority of the citrine-ferritin aggregates displays magnetism and are therefore purified, but do not exhibit strong fluorescence. To compare the two approaches quantitatively, we calculated the purification success (%) by comparing the fluorescence of the MG fraction to the fluorescence of the washed pellet (P) fraction obtained via centrifugation (set to 100%).

This quantification assumed that all citrine-ferritin aggregates that are obtained via centrifugation could in theory be purified using the columns and would display fluorescence, yielding up to 42% purification efficiency for the magnetic column purification method. Moreover, as the magnetic purification method excludes impurities (Figure 5), it can potentially make up for this loss in cases where high purity is preferable over high quantity. In conclusion, the above presented experiments suggest that MPAs represent magnetic protein aggregates that can be solely biologically produced in *E. coli*, without requiring *ex vivo* iron loading.

### 3.3 Extension of the strategy to generate CatMPAs

**FIGURE 6:**
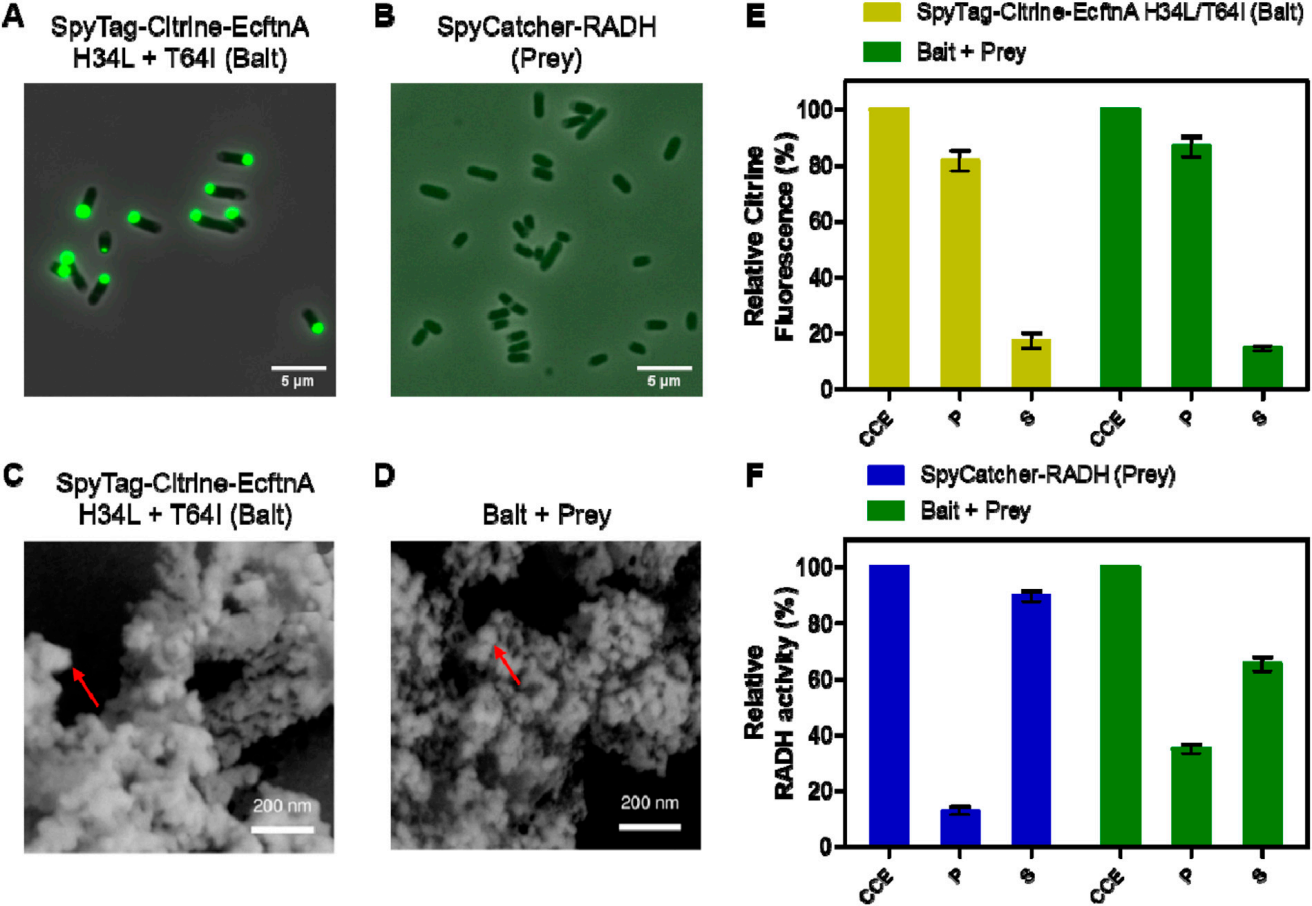
Microscopic analyses and relative fluorescence/activity data for bait and prey constructs. Fluorescence microscopy and phase contrast pictures of live *E. coli* BL21 (DE3) cells overproducing SpyTag-Citrine-EcftnA H34L/T64I (bait) **(A)** and SpyCatcher-RADH (prey) **(B)**. Both panels show fluorescence/phase contrast composite images. Scanning electron microscopy images of lyophilized SpyTag-Citrine-EcftnA H34L/T64I (bait, **(C)**, and the CatMPAs of bait and prey **(D)**. The imaged CatMPA sample was obtained by mixing the crude cell extracts of SpyTag-Citrine-EcftnA H34L/T64I and SpyCatcher-RADH as described above, followed by fractionation and washing steps to obtain the CatMPA containing pellet, which was then lyophilized and imaged. For details, see Methods). The red arrows indicate the smoother, spherical regions which make up the larger MPA aggregate. Relative citrine fluorescence **(E)** and relative RADH activity **(F)** of cell fractions of SpyTag-Citrine-EcftnA H34L/T64I (bait, depicted in yellow), SpyCatcher-RADH (prey, depicted in blue) along with the cell fractions of 1:1 (v/v) mixture of the two constructs (bait + prey, depicted in green). CCE: crude cell extract. P: washed pellet. S: supernatant. Error bars correspond to standard error of the mean obtained from at least three biological replicates.

Next, the magnetic immobilization strategy was further extended as a proof-of-concept to immobilize an alcohol dehydrogenase from *Ralstonia sp.* (RADH) via the CatMPA strategy (Figure 1E). To this end, we used the SpyTag/SpyCatcher technology (Zakeri et al., 2012), which is based on the engineered CnaB2 domain from a *Streptococcus pyogenes* adhesin, where the SpyTag peptide and SpyCatcher protein arising from the split CnaB2 domain can form a spontaneous, irreversible amide bond that can be used to link two proteins together. We implemented the SpyTag/SpyCatcher system to link the insoluble, Citrine-EcftnA H34L/T64I protein fusion (bait) to soluble RADH (prey), to be able to pull RADH into the insoluble fraction. To this end, the genes encoding SpyTag and SpyCatcher were fused to the 5′ of the genes encoding the bait and the prey, respectively (Figure 1D). To check if the presence of the SpyTag infers with the generation of fluorescent aggregates for the bait construct, and to confirm that the presence of the SpyCatcher does not result in the formation of significant amounts of RADH inclusion bodies that would shift the RADH to insoluble fraction for the prey, the live cells overproducing both constructs were evaluated by phase contrast and fluorescence microscopy (Figures 6A,B). The microscopic analyses confirmed that the presence of the SpyTag did not interfere with the formation of insoluble, fluorescent particles for the bait, and SpyCatcher-RADH (prey) showed no particle formation as anticipated. This is further corroborated by SEM analyses (Figure 6C). The morphology of SpyTag-Citrine-EcftnA H34L/T64I MPAs was similar to its counterpart lacking the SpyTag, revealing heterogenous agglomerates, assembled from larger spherical regions as seen for MPAs (Figure 2B).

To link the bait and prey constructs, the strains overproducing SpyTag-Citrine-EcftnA H34L/T64I and SpyCatcher-RADH were cultivated separately, the cells were lysed and their CCEs were mixed in 1:1 (v/v) ratio. The CCE mixture was then incubated at 25°C for 30 min to allow the SpyTag/SpyCatcher interaction to take place, after which the mixed CCE was fractionated into soluble and insoluble fractions. No gross difference in particle morphology was detectable for lyophilized CatMPAs, compared to MPAs by SEM (Figure 6D). Subsequently, fluorescence and RADH activity of the appropriate fractions were determined (for details, see Preparation of cell fractions). The unmixed CCEs of bait and prey constructs were also fractionated to obtain the soluble and insoluble cell fractions, which were tested for fluorescence for the bait construct and RADH activity for the prey (Figure 6). For the bait construct (Figure 6E, yellow bars), citrine fluorescence was detected predominantly in the insoluble fraction (82%), similarly high when compared to the Citrine-EcftnA H34L/T64I construct lacking the SpyTag (Figure 3). For the prey construct, only 13% of the RADH activity could be found in the insoluble fraction (Figure 6F, blue bars). Upon mixing the CCEs of bait and prey constructs, the RADH activity of the insoluble fraction could be increased to 35% of the total RADH activity of the mixture (Figure 6F, green bars), corresponding to an almost 3-fold activity increase in this fraction. This suggests that, RADH could be successfully immobilized on MPAs via the SpyTag/SpyCatcher interaction to yield CatMPAs. In order to exclude that hydrophobic interactions rather than the specific SpyTag/SpyCatcher-based protein-protein interaction, is responsible for the increase of RADH activity for the bait + prey pellet, control experiments were conducted with bait-prey pairs either lacking SpyTag or SpyCatcher. To this end, the CCEs of two negative control pairs; 1) Citrine-EcFtnA H34L/T64I (bait lacking SpyTag) + SpyCatcher-RADH (prey), and 2) SpyTag-Citrine-EcFtnA H34L/T64I (bait) + soluble RADH (prey lacking SpyCatcher) were mixed, incubated and fractionated under the same conditions as described for CatMPA generation. The RADH activity of the pellets derived from these mixtures did not increase significantly when compared to the pellet of the RADH-containing constructs for each case (from 1.66% to 0.89% for the first negative control pair, and from 0.92% to 1.43% for the second pair respectively, see Supplementary Table S6). This proves, the specificity of the CatMPA assembly reaction, relying on covalent SpyTag/SpyCatcher coupling.

Unfortunately, altering bait:prey ratios and incubation times did not result in significantly higher activity in the insoluble fraction, similar to alternative bait and prey constructs tested which harbored the tags at different termini (Supplementary Tables S5 and S6). This suggests that, likely due to the heterogeneous nature of the MPA aggregates (see SEM studies, Figures 2, 6), a further increase of the enzyme load might be restricted by the limited accessibility of the ferritin-displayed SpyTag moiety. A different approach relying on “magnetization” of GFIL8-RADH CatIBs (Ölçücü et al., 2022) by soluble ferritin cages was likewise tested with different SpyTag/SpyCatcher constructs (Supplementary Table S6), however, the SpyTag-Citrine-EcftnA H34L/T64I and SpyCatcher-RADH combination presented here, yielded the best results in terms of relative RADH activity for the pellet derived from the bait + prey mixture (Supplementary Table S6). For instance, the combination of SpyCatcher-EcftnA H34L/T64I and GFIL8-RADH-SpyTag pair, which relied on the magnetization of insoluble GFIL8-RADH CatIBs with soluble ferritin, yielded the highest purification yield (over 97%, Supplementary Table S6), where these magnetized CatIBs could be purified via magnetic columns, similar to MPAs. However, the here presented SpyTag-Citrine-EcftnA H34L/T64I + SpyCatcher-RADH CatMPA pair showed higher RADH activity and aggregation efficiency. The CatMPAs of SpyTag-Citrine-EcftnA H34L/T64I + SpyCatcher-RADH CatMPA pair could also be magnetically purified, albeit with a lowered purification efficiency (9.4%–18.4%, as calculated from the relative citrine fluorescence and RADH activity of the magnetic fraction compared to those of the washed pellet, respectively, Supplementary Table S6) when compared to best performing magnetized CatIBs. The difference in CatMPA purification efficiencies calculated via assays conducted on citrine and RADH indicate different amounts of active protein being present in magnetic and washed pellet fractions, where citrine-ferritins with a higher purification efficiency conversely showing a low citrine fluorescence for the bait fusion. Nevertheless, utilizing a bait-prey approach appears to be a feasible way to generate CatMPAs, and testing different bait-prey constructs and combinations proved beneficial for the optimal implementation of the presented strategy.

**FIGURE 7:**
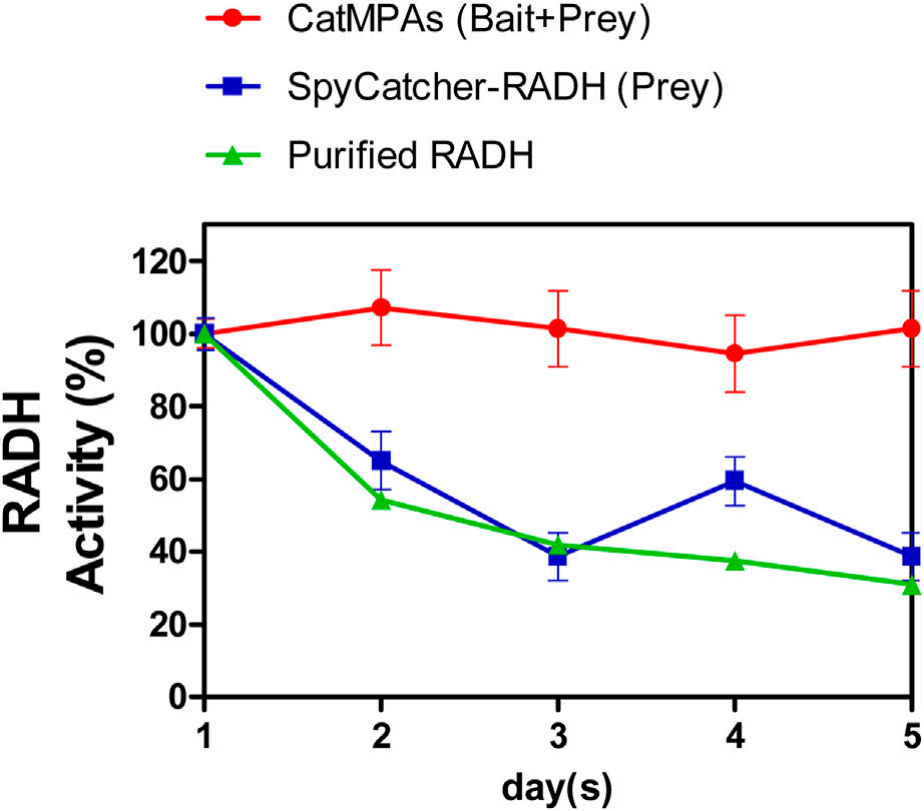
Stability of CatMPAs generated by the SpyTag-Citrine-ferritin (bait) and SpyCatcher-RADH (prey) constructs, compared to the prey construct, and soluble, purified RADH over 5 days. Stability of soluble RADH is taken from (Ölçücü et al., 2022). All error bars depict the standard error of the mean from at least three replicates. Please note that error of measurement of the soluble RADH is below 0.3% and hence, the corresponding error bars are not visible.

Finally, the stability of the CatMPAs was tested by incubating lyophilized CatMPAs, as well as SpyCatcher-RADH, suspended in lysis buffer (50 mM sodium phosphate buffer, 100 mM NaCl, pH 8.0, see methods) at room temperature for 5 days, in the same way as described for RADH CatIBs (Ölçücü et al., 2022). Remarkably, the CatMPAs did not lose any activity over a 5-day period, where the RADH activity at the end of 5 days corresponded to 101.3% of the initial RADH activity detected at day 1 (Figure 7). Within the same time period, the activity of the prey dropped to 38.7% of its initial activity, which is comparable to that of soluble RADH [only 31% after 5 days, under the same conditions (Ölçücü et al., 2022)]. This demonstrates the remarkable stability of CatMPAs, suggesting that CatMPA-based immobilizates represent a promising new enzyme immobilizate for application in biocatalysis and synthetic chemistry.

To the best of our knowledge, the only other modular *in vivo* immobilization method that yields magnetic enzyme immobilizates, not requiring *ex vivo* iron loading, utilizes bacterial magnetosomes, i.e., membrane-enclosed inclusions of magnetite (Fe_3_O_4_), which allow certain magnetotactic bacteria to orient themselves along the geomagnetic field (Mickoleit and Schüler, 2019). While this strategy has been employed widely for the display of proteins on biologically produced magnetic particles, including the modular display of enzymes for biotechnological application (Mittmann et al., 2022), it is at present restricted to magnetotactic bacteria such as *Magnetospirillum magneticum* AMB-1 and *Magnetospirillum gryphiswaldense* MSR-1 and related proteobacteria (Dziuba et al., 2024), whose cultivation is more difficult to scale as compared to *E. coli* (Basit et al., 2020).

## 4 Conclusion

In this study, we successfully generated magnetic protein aggregates (MPAs) by the overproduction of citrine-ferritin fusions, and extended the strategy from human ferritin (Bellapadrona et al., 2015) to wild-type and magnetically enhanced *E. coli* ferritin (Liu et al., 2016) variants, which yielded constructs with superior aggregation efficiencies. Furthermore, we were able to demonstrate the magnetic properties displayed by the MPAs for the first time, and further exploited this property to purify and obtain protein immobilizates of high purity directly from crude cell extracts. To the best of our knowledge, this is the first report describing the generation of fully *in vivo* produced protein aggregates which could be magnetically purified without *ex vivo* iron loading. In proof-of-concept experiments, we generated enzyme-linked magnetic aggregates utilizing the SpyTag/SpyCatcher (Keeble et al., 2017; Keeble and Howarth, 2019) technology to link citrine-ferritin MPAs to an alcohol dehydrogenase (RADH) to produce catalytically-active magnetic protein aggregates (CatMPAs). CatMPAs can simply be obtained by centrifugation or magnetically purified similar to MPAs. Remarkably, CatMPAs showed superior storage stabilities as compared to the soluble and purified RADH. The here presented CatMPA strategy, therefore, can serve as a modular platform for enzyme immobilization, as it allows the immobilization of new targets with minimal to no construct optimization (i.e., only the addition of a SpyCatcher tag to an immobilization target would be necessary), and therefore can be advantageous when compared to existing *in vivo* immobilization methods. As evidenced by these findings, *in vivo* produced MPAs are a promising new immobilization material solely produced by biological means, yielding catalytically-active magnetic protein aggregates (CatMPAs) in a modular fashion. Compared to other *in vivo* produced enzyme immobilizates (e.g., bacterial magnetosomes linked with enzymes (Mittmann et al., 2022), MPAs/CatMPAs can be produced in *E. coli*. With our study, we thus extended the use of ferritin in biotechnology and further diversify the toolbox of (*in vivo*) enzyme immobilization methods. However, further studies on the aggregation mechanism, the general applicability, e.g., extension to other target enzymes and proteins, and on the efficacy of the method, e.g., in terms of target loading efficiency and recyclability of the CatMPAs, would be required to extend the scope of the CatMPA immobilization method presented here.

## Data Availability

The original contributions presented in the study are included in the article/Supplementary Material, further inquiries can be directed to the corresponding author.
